# Light-Modulated Circadian Synaptic Plasticity in the Somatosensory Cortex: Link to Locomotor Activity

**DOI:** 10.3390/ijms252312870

**Published:** 2024-11-29

**Authors:** Małgorzata Jasińska, Ewa Jasek-Gajda, Marek Ziaja, Jan A. Litwin, Grzegorz J. Lis, Elżbieta Pyza

**Affiliations:** 1 Department of Histology, Jagiellonian University Medical College, 31-034 Krakow, Poland; ewa.jasek@uj.edu.pl (E.J.-G.); marek.ziaja@uj.edu.pl (M.Z.); j.a.litwin@uj.edu.pl (J.A.L.); grzegorz.lis@uj.edu.pl (G.J.L.); 2 Department of Cell Biology and Imaging, Institute of Zoology and Biomedical Research, Jagiellonian University, 30-387 Krakow, Poland; elzbieta.pyza@uj.edu.pl

**Keywords:** circadian rhythmicity, synaptic plasticity, influence of light, synaptic protein expression, somatosensory cortex

## Abstract

The circadian clock controls various physiological processes, including synaptic function and neuronal activity, affecting the functioning of the entire organism. Light is an important external factor regulating the day–night cycle. This study examined the effects of the circadian clock and light on synaptic plasticity, and explored how locomotor activity contributes to these processes. We analyzed synaptic protein expression and excitatory synapse density in the somatosensory cortex of mice from four groups exposed to different lighting conditions (LD 12:12, DD, LD 16:8, and LL). Locomotor activity was assessed through individual wheel-running monitoring. To explore daily and circadian changes in synaptic proteins, we performed double-immunofluorescence labeling and laser scanning confocal microscopy imaging, targeting three pairs of presynaptic and postsynaptic proteins (Synaptophysin 1/PSD95, Piccolo/Homer 1, Neurexins/PICK1). Excitatory synapse density was evaluated by co-labeling presynaptic and postsynaptic markers. Our results demonstrated that all the analyzed synaptic proteins exhibited circadian regulation modulated by light. Under constant light conditions, only Piccolo and Homer 1 showed rhythmicity. Locomotor activity was also associated with the circadian clock’s effects on synaptic proteins, showing a stronger connection to changes in postsynaptic protein levels. Excitatory synapse density peaked during the day/subjective day and exhibited an inverse relationship with locomotor activity. Continued light exposure disrupted cyclic changes in synapse density but kept it consistently elevated. These findings underscore the crucial roles of light and locomotor activity in regulating synaptic plasticity.

## 1. Introduction

Synaptic plasticity plays a crucial role in the functioning of the nervous system, enabling the organism to adapt to the external environment and integrate it with internal information. In mammals, circadian rhythms are generated by a central clock (pacemaker) located in the suprachiasmatic nuclei (SCN) of the hypothalamus and modified by peripheral clocks found in various cells of the body. Both the central clock and peripheral clocks significantly influence the regulation of homeostasis and the functioning of numerous biological processes [1,2,3,4].

It is well established that the circadian cycle is closely linked to synaptic plasticity, regulated by the circadian clock. Throughout the 24-h period, changes occur in gene expression [5,6], as well as in the levels and activity of synaptic proteins [7,8]. Furthermore, the number and efficiency of synapses vary throughout the day [9,10,11,12,13]. These time-of-day-dependent changes in the functioning of the nervous system significantly influence learning, memory, and other cognitive functions [14,15].

Daily rhythmicity of different variables can be measured under standard light–dark 12 h: 12 h (LD 12:12) conditions, as the SCN receives light signals from the retina via the retinohypothalamic tract (RHT), allowing the synchronization of the pacemaker and peripheral clocks with the day–night cycle. The endogenous rhythmicity of physiological and molecular processes can be revealed and accurately assessed under constant darkness (DD), which removes the influence of the external temporal cue (light) [16,17]. It can be assumed the circadian clock controls the changes observed during the rest/activity cycle if they occur exclusively under DD or remain consistent under LD 12:12 and DD conditions. However, if changes in any parameter occur only under LD 12:12 conditions or there are discrepancies between the rhythm pattern in LD 12:12 and DD, this suggests that they are influenced by an additional factor, such as light. Locomotor activity should also be considered, as it is influenced by both the circadian clock and light exposure and can affect plasticity in various brain regions [18,19,20].

It has been shown that extending the light phase affects clock gene expression and suppresses changes in synaptic activity in the SCN during subjective night [21,22]. Prolonged light exposure has also been observed to influence circadian changes in the morphology of hippocampal neurons and the rhythmicity of dendritic spine density [23].

Constant light is a highly stressful environment for nocturnal animals, gradually suppressing locomotor activity rhythm and sleep–wake cycle [24,25]. Exposure to constant light weakens the SCN neural network, leading to the desynchronization of clock neurons and disruption of biological process rhythmicity. It results in behavioral arrhythmia or splitting the rhythm of activity and rest, though it does not impair the SCN’s ability to generate circadian rhythms [26]. Additionally, under constant light conditions, deficits in spatial memory and long-term depression induction in the hippocampus have been observed [20,27]. Constant light also diminishes or completely abolishes various synaptic changes seen under light–dark conditions, including alterations in synaptic density, dendritic spine density, and the levels of synaptic proteins [20,28,29].

In the mouse somatosensory cortex, layer 4 (the barrel cortex) contains somatotopic representations of the whiskers on the animal’s snout, which are stimulated during locomotor activity. Due to its clear structural organization and the fact that the barrel cortex is a site of rapid plastic changes, it serves as an excellent target for studies of circadian synaptic plasticity [30,31,32]. Although the somatosensory cortex has no direct connection with the visual pathway, light can influence behavior and physiological processes in mammals through intrinsically photosensitive retinal ganglion cells [33,34,35]. Projections from these cells, via the RHT, reach various brain regions involved in regulating the activity and sleep rhythm and also affect the mechanisms related to the attention system, such as glucocorticoid levels, heart rate, and increased alertness and vigilance [36,37,38,39].

Previous studies show that under LD 12:12 conditions, the number of excitatory synapses in the fourth layer of the mouse somatosensory cortex is higher during the rest phase, i.e., during the day. In DD conditions, no differences in excitatory synapse density are observed between the rest and activity phases [30,40]. These findings suggest that the quantitative changes in excitatory synapses are light-dependent, as their density remains stable across both phases without light cues.

Studies on the circadian rhythmicity of proteins associated with excitatory synapses in the somatosensory cortex are limited. It was found that the mRNA level of the postsynaptic protein Homer 1 increases in rats in the middle of the night (activity phase) under LD 12:12 conditions [32]. Homer proteins are markers of the excitatory synapses and function as the scaffolding proteins within the postsynaptic density of these synapses. They also play an important role in interacting with metabotropic glutamate receptors (mGluRs), highlighting their significance in regulating the function of excitatory synapses [41]. In the superficial layers of the mouse somatosensory cortex, activity-regulated cytoskeleton-associated (Arc) protein shows a higher ratio in the nucleus to the cytoplasm during sleep (rest phase), suggesting that the alpha-amino-3-hydroxy-5-methyl-4-isooxazole-propionic acid receptors (AMPARs) may be downregulated during sleep [31]. Arc is associated with the endocytosis of AMPARs from synapses, leading to decreased synaptic strength [42]. Understanding the changes in proteins associated with various receptors appears essential to our further knowledge of the mechanisms regulating the circadian plasticity of excitatory synapses.

Previous studies on circadian and daily synaptic changes in the mouse primary somatosensory cortex have mainly been focusing on two conditions: LD 12:12 and DD. In this study, we broadened the analysis by introducing additional light conditions, including prolonged photoperiod (LD 16:8) and constant light (LL), to gain a more comprehensive understanding of light’s impact on synaptic dynamics. Furthermore, we expanded the investigation beyond the typical comparison of day and night or subjective day and subjective night by including additional time points, such as the middle of the day/subjective day and the middle of the night/subjective night. This approach provides a deeper insight into changes in protein expression and circadian synaptic plasticity. A detailed analysis of the animals’ locomotor activity was performed under all selected light conditions to better understand the connection between synaptic plasticity and locomotor activity.

In the present study, we used double-immunofluorescence labeling and laser scanning confocal microscopy imaging, which allowed the precise identification of specific presynaptic and postsynaptic proteins related to synaptic functionality in large brain tissue areas. Three pairs of proteins were selected to comprehensively analyze circadian changes in excitatory synapse density and explore potential associations with specific receptors. Our findings offer valuable insights into the complex mechanisms governing excitatory transmission within the context of circadian rhythms.

## 2. Results

### 2.1. Locomotor Activity of Animals

**Figure 1:**
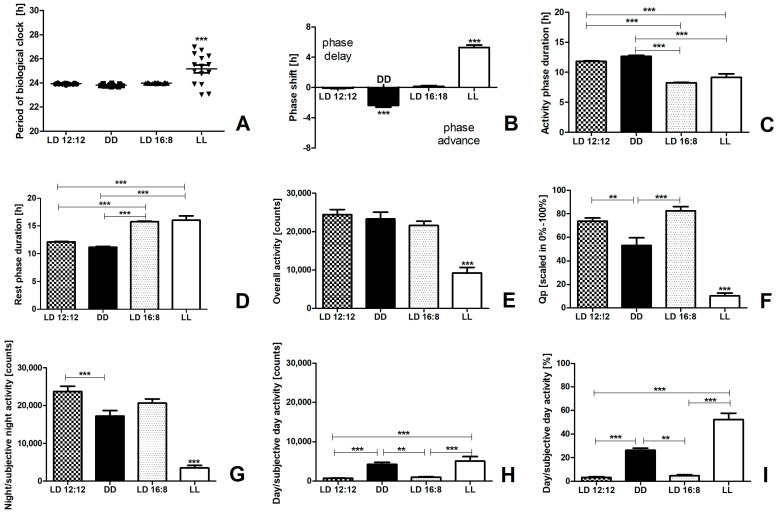
Analysis of daily and circadian rhythmicity. (**A**) *Tau*—period of daily/circadian rhythm [h]; (**B**) *Delta*—shift (phase advance or phase delay) of the activity onset [h]; (**C**) *Alpha*—duration of activity phase [h]; (**D**) *Rho*—duration of rest phase; (**E**) overall activity [wheel revolutions/day]; (**F**) Qp—robustness of circadian rhythm [%]; (**G**) night/subjective night activity [wheel revolutions/day]; (**H**) day/subjective day activity [wheel revolutions/day]; and (**I**) percentage of activity during the day/subjective day to total activity [%]. The experimental light conditions: light–dark 12 h:12 h (LD 12:12), constant darkness (DD), long photoperiod LD 16 h:8 h (LD 16:8), and constant light (LL). The graphs show means ± SEM (one-way ANOVA; *** *p* < 0.001 and ** *p* < 0.01). The asterisks located directly above the bars signify that the difference applies to all groups.

We conducted a comprehensive analysis of locomotor activity across examined groups (LD 12:12—12 h of light and 12 h of darkness, DD—constant darkness, LD 16:8—16 h of light and 8 h of darkness, and LL—constant light) to consider potential differences related to the conditions in which the animals were housed. All the analyzed parameters of locomotor activity are listed in Table 1, and the analyzed results are shown in Figure 1.

#### Analysis of Locomotor Activity Parameters

*Tau*. The period of daily/circadian rhythm was calculated using methods described in the Section 4.2. Regardless of the algorithm used, the LL group exhibited a longer period compared to the other groups: 1.22 h longer than the LD 12:12 group, 1.34 h longer than the DD group, and 1.17 h longer than the LD 16:8 group (*p* < 0.0001) (Figure 1A). There was also a greater data scatter within the LL group.

*Delta*. No statistically significant differences in the onset of activity were observed between the LD 12:12 and LD 16:8 groups (Figure 1B). In the LD 16:8 group, the lights were turned off four hours later (prolonging the light phase to 16 h) compared to the acclimation period. This resulted in an initial acute delay in the onset of activity by 4 h, but the rhythm stabilized within 24 h. Only minor changes were observed (1.3 ± 3.2 min of delay of the onset activity), comparable to those observed in the LD 12:12 group (1.1 ± 1.6 min of advance of the onset of activity). In contrast, significant changes were seen in the groups exposed to constant conditions (DD and LL groups). The DD group showed a phase advance of 2.37 ± 0.86 h compared with the LD 12:12 group, with an average advance of 19.0 ± 7.2 min per period (*p* < 0.001). Conversely, a pronounced phase delay of 5.29 ± 1.0 h was observed compared to the LD 12:12 group, with delay of 46.3 ± 9.0 min at the onset of the activity phase (*p* < 0.001).

*Alpha*. The duration of the activity phase did not differ significantly between the LD 12:12 and DD groups (DD—12.7 ± 0.16 h; LD 12:12—11.8 ± 0.08 h) (Figure 1C). In contrast, the LL group showed a decrease in *alpha* by over 22% relative to the LD 12:12 group (LL—9.1 ± 0.59 h), while the LD 16:8 group had the shortest *alpha*, with a decrease of about 30% compared to the LD 12:12 group (LD 16:8—8.2 ± 0.08 h; *p* < 0.0001).

*Rho*. The duration of the rest phase was significantly longer in LD 16:8 and LL than in the other groups (*p* < 0.001) (Figure 1D).

**Figure 2:**
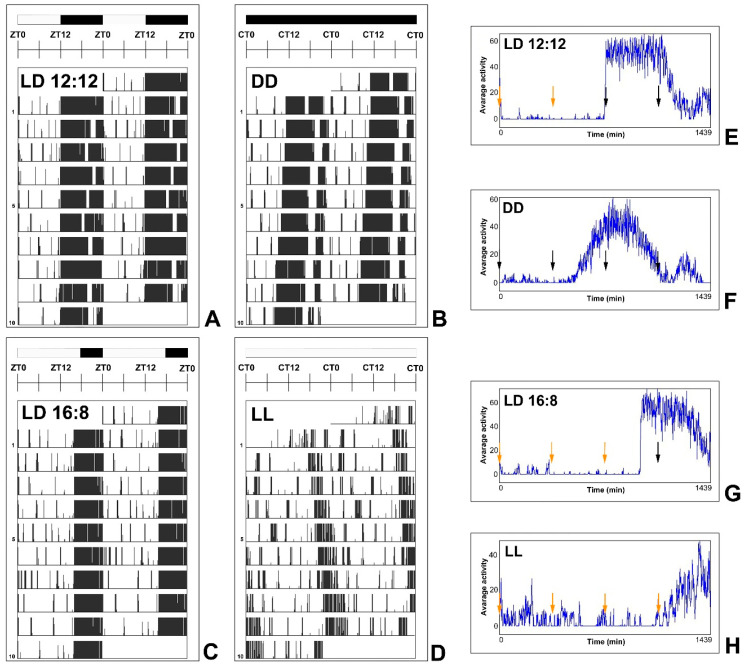
Daily and circadian locomotor activity. The left panel (**A**–**D**) shows representative double-plotted actograms of mice running-wheel activity under different conditions. The right panel (**E**–**H**) displays representative periodograms (average waveform of activity) showing mouse locomotor activity as measured over 10 days under different conditions. The time points at which the mice were sacrificed are indicated by the arrows—yellow arrow if in light, black if in darkness. The experimental light conditions: light–dark 12 h:12 h (LD 12:12), constant darkness (DD), long photoperiod LD 16 h:8 h (LD 16:8), and constant light (LL).

Overall activity. Mice from the LL group showed more than 62% lower overall activity compared to the LD 12:12 group (*p* < 0.0001) (Figure 1E). The result of overall activity in the LD 16:8 group did not differ from the LD 12:12 and DD groups. The *alpha* of the LD 16:8 group was shorter by an average of 3.6 h compared to the LD 12:12 group and, even more, by 4.4 h compared to the DD group (Figure 1C). Since there were breaks during the highest activity (see Figure 2A,B) and the length of these breaks (LD 12:12—1.46 ± 0.14 h; DD—2.34 ± 0.16 h; LD 16:8—0.29 ± 0.05 h) correlated with the *alpha* in the LD 12:12 and DD groups (Supplementary Materials, Figure S1A,B), we decided to investigate whether these breaks could explain the situation. Our analysis revealed that even after subtracting the time spent by the animals on activity breaks, the activity period was still significantly shorter in the LD 16:8 and LL groups compared to the other groups (Supplementary Materials, Figure S1C), suggesting more intense activity of animals in the LD 16:8 group during a shorter period. No significant differences in overall activity levels were observed between the other groups.

Robustness of daily/circadian rhythm—Qp. The LD 12:12 and LD 16:8 groups showed similar % Qp values, both above 70%, indicating a high level of rhythm stability. In contrast, the DD group exhibited lower stability (53.2 ± 6.14%), while the LL group showed the lowest stability level (10.2 ± 2.48%; *p* < 0.001) (Figure 1F).

Night (ZT12-ZT24)/subjective night activity (CT12-CT24). The locomotor activity of the animals during the subjective night differed in groups kept under constant conditions (the DD and LL groups) compared to the activity in the LD 12:12 group (Figure 1G). The mice from the LL group were characterized by significantly lower activity during the subjective night compared to the other groups (85% lower activity than in the LD 12:12 group; *p* < 0.0001). Similarly, the DD group had over 27% lower activity than the mice from the LD 12:12 group.

Day (ZT0-ZT12)/subjective day activity (CT0-CT12). The subjective day activity (Figure 1H) was higher in the LL group than in both the LD 12:12 and LD 16:8 groups (seven times higher compared to day activity in the LD 12:12 group and five times higher than day activity in the LD 16:8 group; *p* < 0.0001). Similarly, subjective day activity in the DD group was three times higher than in the LD 12:12 group and twice as high as in the LD 16:8 group, respectively. There was no statistical difference between the LL and DD groups. The lowest percentages of day activity in relation to overall activity were observed in the LD 12:12 (3.3 ± 0.69%) and LD 16:8 groups (4.8 ± 0.75%) (Figure 1I). In contrast, the DD group exhibited a significantly higher percentage of subjective day activity at 26.3 ± 1.75% (*p* < 0.01). The LL group displayed the highest percentage of subjective day activity, 52.2 ± 5.31% higher than the LD 12:12 group (*p* < 0.001).

Additional data regarding activity during the activity and rest phases in each animal group can be found in the Supplementary Materials (Figure S1D,E).

### 2.2. Immunohistochemical Results

**Figure 3:**
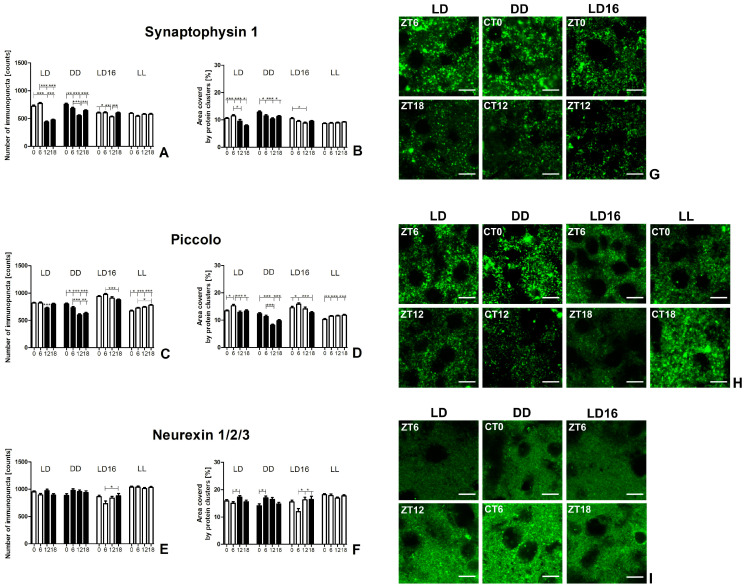
Daily and circadian changes in the expression of presynaptic proteins. The left panel shows the number of presynaptic protein immunopuncta and the area covered by protein (**A**,**B**) Synaptophysin 1, (**C**,**D**) Piccolo, (**E**,**F**) Neurexin 1/2/3, throughout the day–night or the subjective day–night cycle under different conditions. The graphs show means ± SEM (one-way ANOVA; *** *p* < 0.001, ** *p* < 0.01, * *p* < 0.05). The asterisks located directly above the bars signify that the difference applies to all time points within the group. The right panel displays images illustrating the immunopositive reaction for respective presynaptic proteins, highlighting the minimum and maximum values observed in different conditions where statistically significant differences were observed between individual time points: (**G**) Synaptophysin 1, (**H**) Piccolo, (**I**) Neurexin 1/2/3. Scale bar, 10 µm. The experimental light conditions are as follows: light–dark 12 h:12 h (LD), constant darkness (DD), prolonged light LD 16 h:8 h (LD16), and constant light (LL). Time points are defined as follows: 0—ZT0/CT0: the beginning of the day/subjective day; 6—ZT6/CT6: the middle of the day/subjective day; 12—ZT12/CT12: the beginning of the night/subjective night; 18—ZT18/CT18: the middle of the night/subjective night.

**Figure 4:**
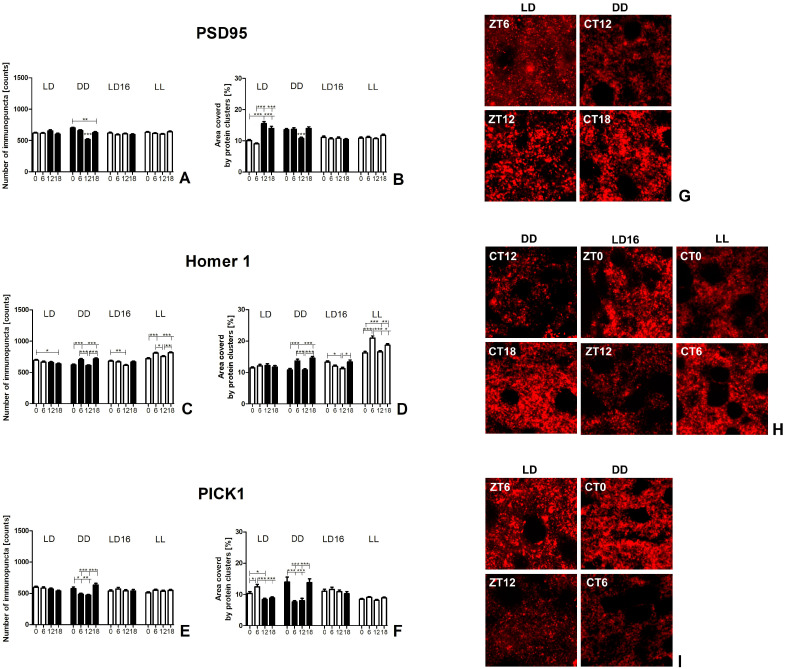
Daily and circadian changes in the expression of postsynaptic proteins. The left panel shows the number of postsynaptic protein immunopuncta and the area covered by protein (**A**,**B**) PSD95, (**C**,**D**) Homer 1, and (**E**,**F**) PICK1 throughout the day–night or the subjective day–night cycle under different conditions. The graphs show means ± SEM (one-way ANOVA; *** *p* < 0.001, ** *p* < 0.01, * *p* < 0.05). The asterisks located directly above the bars signify that the difference applies to all time points within the group. The right panel displays images illustrating the immunopositive reaction for respective presynaptic proteins, highlighting the minimum and maximum values observed in different conditions where statistically significant differences were observed between individual time points: (**G**) PSD95, (**H**) Homer 1, (**I**) PICK1. Scale bar, 10 µm. The experimental light conditions: light–dark 12 h:12 h (LD), constant darkness (DD), prolonged light LD 16 h:8 h (LD16), and constant light (LL). Time points are defined as follows: 0—ZT0/CT0: the beginning of the day/subjective day; 6—ZT6/CT6: the middle of the day/subjective day; 12—ZT12/CT12: the beginning of the night/subjective night; 18—ZT18/CT18: the middle of the night/subjective night.

Two different parameters related to the level of synaptic protein expression were analyzed: the number of distinct points (immunopuncta) representing separate protein clusters and the area covered by protein clusters (Figure 3 and Figure 4).

It is worth noting the interaction between the above parameters provides additional information. A simultaneous increase or decrease in both allows for a more comprehensive confirmation of changes in the actual level of protein expression. An increase in the area covered by the protein without significant changes in the number of immunopuncta suggests an increase in protein expression level, but only within pre-existing protein clusters. Conversely, a change in the number of immunopuncta without a corresponding modification in the area covered by the protein indicates dispersion or aggregation, reflecting protein redistribution in the analyzed region.

Data were collected at 6-h intervals, thus, we defined four time points across the day–night cycle or subjective day–subjective night cycle (Figure 2).

#### 2.2.1. Presynaptic Protein Expression

Synaptophysin 1 (Syp1). Daily and circadian variations in the number of Syp1+ immunopuncta and the area covered by the protein were observed in the LD 12:12, DD, and LD 16:8 groups, though no significant changes were detected in the LL group for either parameter (number: *p* = 0.076; area: *p* = 0.390) (see Table 2, Figure 3A,B,G).

In the LD 12:12 group, the number of Syp1+ immunopuncta increased significantly during the day (*p* < 0.0001). Specifically, there was a rise by 64.8% at the beginning of the day (ZT0) and by 76.2% in the middle of the day (ZT6) compared to the beginning of the night (ZT12). Additionally, compared to the middle of the night (ZT18), the increases were by 52.2% at ZT0 and 62.7% at ZT6. Similar changes were found in the area covered by Syp1, although the day–night differences were less pronounced than those in Syp1+ immunopuncta (*p* < 0.0001). The smallest Syp1-covered area was observed in the middle of the night (ZT18), with an increase by 46.1% to its peak in the middle of the day (ZT6). The consistent changes in both parameters under light–dark conditions indicate that Syp1 expression level cyclically decreased at night and increased during the day, reaching a maximum in the middle of the day.

In the DD group, the Syp1 expression level also remained elevated during the subjective day. The lowest number of immunopuncta was found at the beginning of the subjective night (CT12), followed by a 37.2% increase, reaching a peak at the beginning of the subjective day (CT0; *p* < 0.0001). The area covered by Syp1 was significantly greater at the beginning of the subjective day (CT0) compared to at other time points, with increases by 12.3% over CT6, 24.1% over CT12, and 14.2% over CT18 (*p* = 0.0001). Both parameters consistently indicate an increased protein expression level at the beginning of the subjective day in the DD group. On the other hand, a noticeable decrease in the Syp1+ immunopuncta at the beginning of the subjective night in the DD group, without significant changes in the area covered by the protein, suggests a shift in protein distribution, leading to fewer but larger protein clusters.

A similar decline in the number of immunopuncta at ZT12, as observed in the DD group, was also evident in the LD 16:8 group (*p* = 0.002), with a decrease of over 11% compared to all other time points. Furthermore, the area covered by Syp1 was smaller at the ZT12, with a reduction by 15.1%, but only in comparison to the beginning of the day (ZT0; *p* = 0.004). The increase in the area covered by Syp1+ suggests an increase in protein expression level at the beginning of the day in the LD 16:8 group. However, due to the lack of visible changes in the number of immunopuncta, the increase in Syp1 expression level likely reflected only the enlargement of existing protein clusters.

Piccolo. Daily and circadian changes in Piccolo expression levels were found in all groups, including in the LL group (see Table 1, Figure 3C,D,H).

In the LD 12:12 group, the beginning of the night (ZT12) was characterized by a decrease in Piccolo immunopuncta, with counts at ZT12 being 11.2% less than at ZT0, 11.4% less than at ZT6, and 9.0% less than at ZT18 (*p* < 0.0001). Despite this decrease, there was no corresponding reduction in the area covered by the protein. However, Piccolo covered the largest area in the middle of the day (ZT6), with ZT6 values exceeding those at ZT0 by 13.7%, ZT12 by 18.8%, and ZT18 by 14.2% (*p* = 0.0008). These results indicate an increase in Piccolo expression level in previously existing locations in the middle of the day and greater aggregation of Piccolo at the beginning of the night.

In the DD group, an increase in the number of immunopuncta was observed during the subjective day compared to the subjective night, with the highest levels at the beginning of the subjective day (CT0) and the lowest at the beginning of the subjective night (CT12; *p* < 0.0001). At CT0, the number of Piccolo immunopuncta was 33.6% higher than at the beginning of the subjective night (CT12) and 27.8% higher than in the middle of the subjective night (CT18). Additionally, in the middle of the subjective day (CT6), the number of immunopuncta was 21.7% and 16.4% higher than at the beginning of the subjective night (CT12) and in the middle of the subjective night (CT18), respectively. The difference between the beginning (CT0) and the middle of the subjective day (CT6) was small but statistically significant, with an increase of 9.8% at the beginning of the subjective day. These changes in the number of immunopuncta were accompanied by similar changes in the area covered by Piccolo (*p* < 0.0001). At the beginning of the subjective day (CT0), Piccolo covered an area over 50% larger compared to the beginning of the subjective night (CT12) and 25% larger compared to the middle of the subjective night (CT18). Moreover, the area covered by Piccolo was 37.6% larger in the middle of the subjective day (CT6) than at the beginning of the subjective night (CT12). These results consistently indicate differences in Piccolo expression levels between the subjective day, where the expression level was higher, and the subjective night.

In the LD 16:8 group, the number of immunopuncta was 11.0% higher 6 h after the beginning of the day (ZT6) compared to the night (ZT18; *p* = 0.001). A similar effect was observed in the area covered by Piccolo, with a difference of 24.2% (*p* = 0.0001). Additionally, the area covered by Piccolo was 13.9% larger at the beginning of the day (ZT0) than at ZT18. These data indicate an increase in Piccolo expression level during the day in the LD 16:8 group when compared to ZT18, although at the beginning of the day, the increase in expression level was limited to already existing clusters.

In the LL group, decreases in both analyzed parameters were observed at the beginning of the subjective day (CT0). Specifically, the number of Piccolo immunopuncta at CT0 was 7.3% lower than in the middle of the subjective day (CT6), 9.3% lower than at the beginning of the subjective night (CT12), and 13.2% lower than in the middle of the subjective night (CT18; *p* < 0.0001). Similarly, the area covered by Piccolo at CT0 was 10.5% less than at CT6, 11.6% less than at CT12, and 13.1% less than at CT18 (*p* < 0.0001). These findings indicate a decrease in Piccolo expression level at the beginning of the subjective day. Additionally, the number of Piccolo immunopuncta was 6.4% lower in the middle of the subjective day (CT6) than in the middle of the subjective night (CT18).

Neurexin 1/2/3 (NRXNs). NRXNs exhibited slightly smaller daily and circadian fluctuations in both immunopuncta parameters compared to other presynaptic proteins (see Table 2, Figure 3E,F,I). In the LL group, no significant changes were observed in either parameter (number: *p* = 0.722; area: *p* = 0.193), and there were no modifications in the number of NRXN+ immunopuncta in the DD group (*p* = 0.141) across the subjective day–subjective night cycle. Moreover, no significant changes were found between specific time points in the number of NRXN+ immunopuncta in the LD 12:12 group, even though some variability within the group appeared to be present (*p* = 0.041).

An increase of 15.2% in the area covered by NRXNs was observed at the beginning of the night (ZT12) compared to the middle of the day (ZT6) in the LD 12:12 group (*p* = 0.023) and 20.8% in the middle (CT6) compared to the beginning of the subjective day (CT0) in the DD group (*p* = 0.009). Due to the lack of changes in the number of immunopuncta, the observed increases in expression level concerned only the pre-existing protein clusters.

In the LD 16:8 group, the area covered by NRXNs was the smallest at ZT6 compared to all other time points. At ZT6, NRXNs covered 22.9% less area than at the beginning of the day (ZT0), 26.5% less than at ZT12, and 27.0% less than at ZT18 (*p* = 0.009). Additionally, there were 16.5% fewer NRXN+ immunopuncta in the day (ZT6), though a significant difference was only found in comparison with ZT18 (*p* = 0.042). These results indicate a decrease in NRXNs expression level 6 h after the beginning of the day.

#### 2.2.2. Postsynaptic Protein Expression

Postsynaptic density protein 95 (PSD95). The PSD95 expression level showed changes under light–dark conditions and in the DD group. No significant changes were observed in the LD 16:8 (number: *p* = 0.429; area: *p* = 0.678) and LL (number: *p* = 0.058; area: *p* = 0.142) groups (see Table 2, Figure 4A,B,G).

In the LD 12:12 group, an increase of 54.4% and 71.4% was observed at the beginning of the night (ZT12), and 38.6% and 53.9% in the middle of the night (ZT18), in the area covered by PSD95, compared to the beginning of the day (ZT0) and the middle of the day (ZT6), respectively (*p* < 0.0001). However, this increase was not accompanied by a rise in immunopuncta (*p* = 0.100). These results indicate an increase in PSD95 expression level at night compared to the day, but only in pre-existing protein clusters.

In contrast, in the DD group, both the area covered by the protein and the number of immunopuncta decreased at the beginning of the subjective night. Specifically, the number of immunopuncta at CT12 was 26.3% lower than at the beginning of the subjective day (CT0), 21.6% lower than in the middle of the subjective day (CT6), and 17.7% lower than in the middle of the subjective night (CT18; *p* < 0.0001). Similarly, the area covered by PSD95 at the beginning of the subjective night (CT12) was 20.3% lower than at CT0, 21.4% lower than at CT6, and 22.8% lower than at CT18 (*p* < 0.0001). Additionally, an increase of 11.7% in the number of immunopuncta was observed at the beginning of the subjective day (CT0) but only when compared to the middle of the subjective night (CT18), and this increase was not accompanied by a rise in the area covered by PSD95. These results clearly showed a marked decrease in expression level at the beginning of the subjective night under DD conditions.

Homer 1. Rhythmicity in the Homer 1 expression level was observed in the DD, LD 16:8, and LL groups. However, in the LD 12:12 group, the Homer 1 expression level did not change throughout the day–night cycle; instead, its distribution was modified (see Table 2, Figure 4C,D,H).

In the LD 12:12 group, a 9% increase in the number of Homer 1 immunopuncta was observed at the beginning of the subjective day (ZT0) compared to the middle of the subjective night (ZT18; *p* = 0.028). Since no corresponding increase in the area covered by Homer 1 was detected (*p* = 0.596), this suggests a greater dispersion of the protein during the day.

In the DD group, the number of Homer 1 immunopuncta increased by 14.1% in the middle of the subjective day (CT6) compared to the beginning of the subjective day (CT0), and by 15.6% compared to the beginning of the subjective night (CT12; *p* < 0.0001). Additionally, the number of immunopuncta increased by 15.7% and 17.3% in the middle of the subjective night (CT18) compared to the beginning of the subjective day (CT0) and subjective night (CT12), respectively. These increases were accompanied by a 26.5% expansion of the area covered by Homer 1 in the middle of the subjective day (CT6) compared to the beginning of the subjective day (CT0), and 26.1% compared to the beginning of the subjective night (CT12; *p* < 0.0001). Moreover, the area covered by Homer 1 showed an additional peak in the middle of the subjective night (CT18), with increases of 34.8% and 34.4% compared to the beginning of the subjective day (CT0) and night (CT12), respectively. These results clearly demonstrate that Homer 1 exhibited two peaks in expression level during the 24-h cycle: one in the middle of the subjective day and another in the middle of the subjective night.

In the LD 16:8 group, 11.1% more immunopuncta were observed at the beginning of the day (ZT0) than at ZT12 (*p* = 0.009). The area covered by Homer 1 was also 18.5% greater at ZT0 compared to ZT12 (*p* = 0.011). This reduction in area at ZT12 was followed by an 18.7% increase in the night (ZT18). Thus, under the long photoperiod, an increase in Homer 1 expression level was observed at night (ZT18), persisting until the beginning of the day.

Similar to the DD group, the LL group exhibited an increase in the number of immunopuncta, rising by 12.3% and 7.4% in the middle of the subjective day (CT6), and by 13.2% and 8.2% in the middle of the subjective night (CT18), compared to the beginning of the subjective day (CT0) and night (CT12), respectively (*p* < 0.0001). Additionally, the area covered by Homer 1 expanded by 28.6% and 26.3% in the middle of the subjective day, and by 14.9% and 12.8% in the middle of the subjective night, compared to the beginning of the subjective day (CT0) and night (CT12), respectively (*p* < 0.0001). The consistency of changes in both Homer 1 immunopuncta parameters clearly showed the presence of two peaks in Homer 1 expression levels in the middle of both the subjective day and night.

Protein interacting with C alpha kinase 1 (PICK1). PICK1 showed daily rhythmicity in protein expression levels in the LD 12:12 group, while circadian changes were observed only in the DD group (see Table 2, Figure 4E,F,I). The analysis did not reveal any changes in the number of immunopuncta or the area covered by PICK1 in the LD 16:8 group (number: *p* = 0.746, area: *p* = 0.605) or the LL group (number: *p* = 0.189, area: *p* = 0.080).

In the LD 12:12 group, the area covered by protein increased during the day compared to the night, with 23.3% and 16.8% more coverage at the beginning of the day (ZT0), and 48.1% and 40.4% more coverage in the middle of the day (ZT6), compared to the beginning (ZT12) and middle of the night (ZT18), respectively (*p* < 0.0001). However, there were no significant changes in the number of PICK1+ immunopuncta between different time points (*p* = 0.099). This indicates an increased protein expression level within pre-existing protein clusters during the day.

A corresponding increase in the number of PICK1+ immunopuncta and the area covered by PICK1, indicating an increase in protein expression level and the formation of new protein clusters, was observed at the beginning of the subjective day (CT0) in the DD group. There were 18.5% and 23.2% more immunopuncta at CT0 than in the middle of the subjective day (CT6), and at the beginning of the subjective night (CT12), respectively, and the area was 84.5% and 75.2% greater at CT0 than at CT6 and CT12, respectively (*p* < 0.0001). Additionally, in the middle of the subjective night (CT18) in the DD group, there were 30.2% and 35.3% more immunopuncta at CT18 than in the middle of the subjective day (CT6) and the beginning of the subjective night (CT12), respectively, and the area was 81.7% and 72.4% greater at CT18 than at CT6 and CT12, respectively. These results suggest that the PICK1 expression level increased under DD conditions in the middle of the subjective night and continued into the beginning of the subjective day.

#### 2.2.3. Density of the Excitatory Synapses

Changes in the number of co-labeled immunopuncta, reflecting the colocalization of presynaptic and postsynaptic proteins, provide detailed information about the alterations in the number of excitatory synapses across daily or circadian cycles [43]. All examined postsynaptic proteins are components of excitatory synapses and are directly or indirectly associated with different types of glutamatergic receptors.

**Figure 5:**
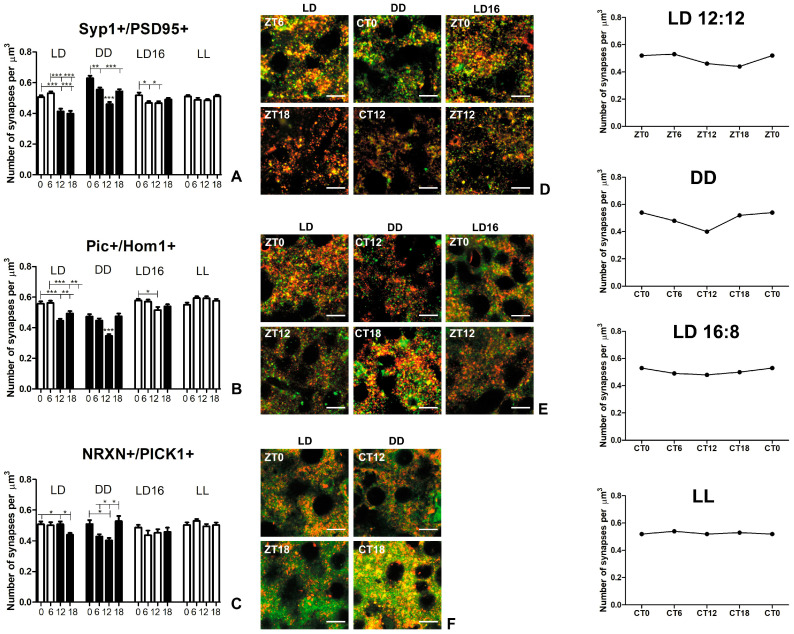
Daily and circadian changes in excitatory synapses. The left panel shows the density of excitatory synapses throughout the day–night or the subjective day–night cycle under different conditions. The synaptic protein pairs are (**A**) Synaptophysin 1—PSD95 (Syp1+/PSD95+), (**B**) Piccolo—Homer 1 (Pic+/Hom1+), and (**C**) Neurexin 1/2/3—PICK1 (NRXN+/PICK1+). The graphs show means ± SEM (one-way ANOVA; *** *p* < 0.001, ** *p* < 0.01, * *p* < 0.05). The asterisks located directly above the bars signify that the difference applies to all time points within the group. The photomicrographs in the middle panel display the double-immunopositive reaction (yellow) for respective presynaptic (green) and postsynaptic (red) proteins, highlighting the time points for which the statistically significant differences were found in particular conditions. (**D**) Syp1+/PSD95+, (**E**) Pic+/Hom1+, (**F**) NRXN+/PICK1+. Scale bar, 10 µm. The right panel shows the averaged synapse density waveform, calculated across all protein pairs, for the different conditions. The experimental light conditions: light–dark 12 h:12 h (LD or LD12:12), constant darkness (DD), prolonged light LD 16 h:8 h (LD16 or LD 16:8), and constant light (LL). Time points are defined as follows: 0—ZT0/CT0: the beginning of the day/subjective day; 6—ZT6/CT6: the middle of the day/subjective day; 12—ZT12/CT12: the beginning of the night/subjective night; 18—ZT18/CT18: the middle of the night/subjective night.

Despite the fact that two of the analyzed proteins—presynaptic Piccolo and postsynaptic Homer 1—exhibited circadian changes in expression level under constant light conditions, no changes were observed in the density of co-labeled immunopuncta in the LL group (see Table 3, Figure 5).

Synapse Syp1+/PSD95+ (synaptophysin 1—PSD95 double-immunopositivity). A higher density of Syp1+/PSD95+ synapses was observed during the day compared to the night in the LD 12:12 group (Figure 5A,D). Specifically, there were 22.5% and 26.7% more synapses at the beginning of the day (ZT0), and 28.9% and 33.3% more in the middle of the day (ZT6), compared to the beginning (ZT12) and middle (ZT18) of the night, respectively (*p* < 0.0001).

In the DD group, the density of Syp1+/PSD95+ synapses decreased to a minimum at the beginning of the subjective night (CT12) and then increased to a maximum at the beginning of the subjective day (CT0). At this peak (CT0), the density of Syp1+/PSD95+ synapses increased by 13.5% compared to the middle of the subjective day (CT6), 36.7% compared to the beginning of the subjective night (CT12), and 15.8% compared to the middle of the subjective night (CT18; *p* < 0.0001). The density remained similar during the middle of the subjective day and night.

In the LD 16:8 group, the highest density of Syp1+/PSD95+ synapses was noted at the beginning of the day (ZT0), with 10.5% more than at ZT6, 10.9% more than at ZT12 and 5.5% more than at ZT18 (*p* < 0.0001).

Synapse Pic+/Hom1+ (Piccolo—Homer 1 double-immunopositivity). The LD 12:12 group exhibited a significantly greater density of Pic+/Hom1+ synapses during the day compared to the night (*p* < 0.0001) (Figure 5B,E). Specifically, at the beginning of the day (ZT0), the density was 25.0% higher than at the beginning of the night (ZT12) and 12.9% higher than in the middle of the night (ZT18). Similarly, in the middle of the day (ZT6), the density of Pic+/Hom1+ synapses was 26.1% higher than at the beginning (ZT12) and 13.9% higher than in the middle of the night (ZT18).

The DD group showed a significantly lower density of Pic+/Hom1+ synapses at the beginning of the subjective night (CT12) compared to the other time points (*p* < 0.0001). Specifically, at CT12, the density was 26.6% lower than at the beginning of the subjective day (CT0), 22.5% lower than in the middle of the subjective day (CT6), and 26.8% lower than in the middle of the subjective night (CT18).

Similarly, in the LD 16:8 group, a reduced density of Pic+/Hom1+ synapses was observed at ZT12, with a 10.8% decrease compared to the beginning of the day (ZT0; *p* = 0.012).

Synapse NRXN+/PICK1+ (neurexin 1/2/3—PICK1 double-immunopositivity). In the LD 12:12 group, the lowest density of NRXN+/PICK1+ synapses was observed in the middle of the night (ZT18), with density at ZT18 being 13.4% lower than at the beginning of the day (ZT0), 12.3% lower than in the middle of the day (ZT6), and 13.4% lower than at the beginning of the night (ZT12; *p* = 0.011) (Figure 5C,F).

In contrast, the DD group showed the highest density of NRXN+/PICK1+ synapses in the middle of the subjective night (CT18), with 23.0% higher than in the middle of the subjective day (CT6) and 30.8% higher than at the beginning of the subjective night (CT12; *p* = 0.0001). Additionally, the density of NRXN+/PICK1+ synapses at the beginning of the subjective night (CT12) was significantly lower by 21.0% compared to the beginning of the subjective day (CT0).

No significant changes were observed in the LD16:8 group (*p* = 0.523).

General patterns of circadian changes in excitatory synapse density. In the DD group, the pattern of circadian changes in excitatory synapse density remained stable, with a minimum at the beginning of the subjective night, regardless of which pairs of presynaptic and postsynaptic proteins were used to visualize the synapses (Figure 5—right panel). During the subjective night, excitatory synapse density gradually increased, reaching a peak in the middle of the night, which was maintained at the beginning of the subjective day. Subsequently, the excitatory synapse density decreased until it reached a minimum.

In the LD 12:12 group, a similar pattern of daily excitatory synapse density changes was observed as in the DD group, but with sharper phase distinctions—daytime was characterized by higher excitatory synapse density, while nighttime showed a lower level. The smallest changes throughout the day–night cycle were observed in NRXN+/PICK1+ synapses, where only a clear minimum density was detected in the middle of the night.

In the LD 16:8 group, a slight decrease in excitatory synapse density was still observed at ZT12, followed by an increase at the beginning of the day. In the LL group, excitatory synapse density remained consistently high, with no significant changes observed. This demonstrates that extended exposure to light reduced or eliminated circadian changes in excitatory synapse density.

#### 2.2.4. Participation of Synaptic Proteins in Excitatory Synapses

**Figure 6:**
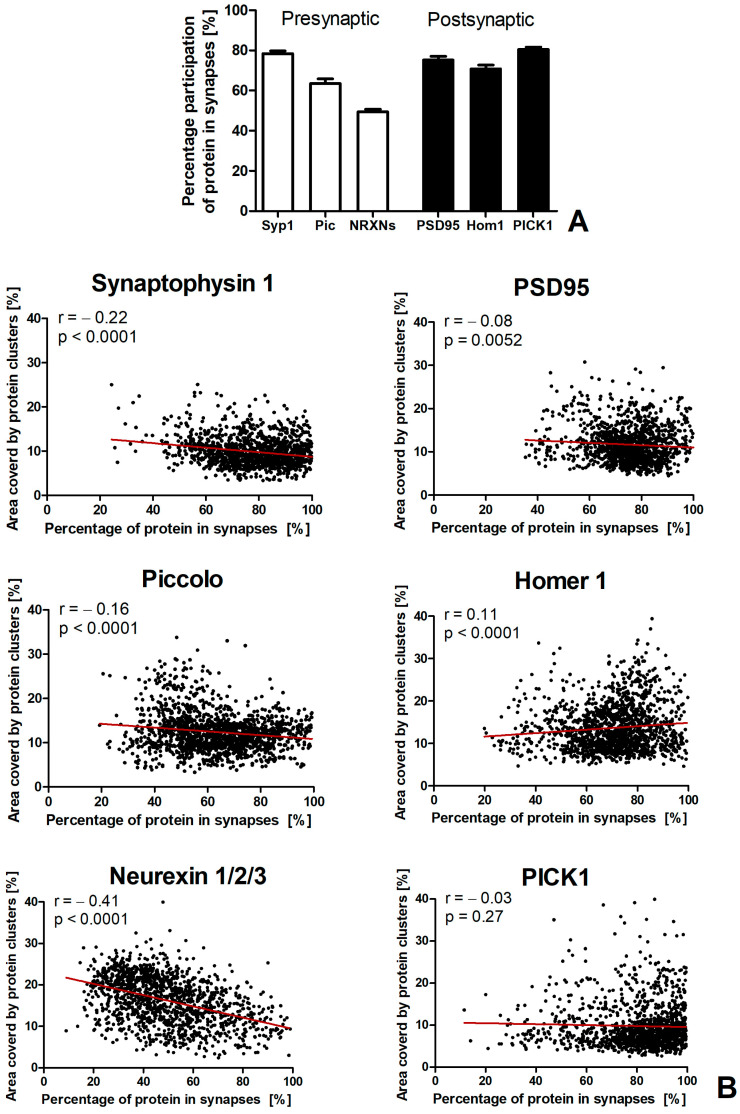
Participation of synaptic proteins in excitatory synapses. (**A**) Percentage participation of presynaptic and postsynaptic proteins in excitatory synapses. (**B**) Correlation between the area covered by a protein and the percentage participation of that protein in excitatory synapses for respective presynaptic (left column) and postsynaptic proteins (right column). Syp 1, Synaptophysin 1; Pic, Piccolo; NRXNs, Neurexin 1/2/3; Hom1, Homer 1; r, Pearson correlation coefficient.

The average percentage content of synaptic proteins in excitatory synapses was approximately 70–80%, except for Piccolo, which barely exceeded 60%, and NRXNs, whose average participation in synapses was slightly below 50% (Figure 6 and Supplementary Materials, Figure S3).

Interestingly, we found a negative correlation between the percentage participation of each presynaptic protein and the number of immunopuncta (*p* < 0.0001), as well as the area covered by those proteins (*p* < 0.0001). This means that, as the number of immunopuncta and the area covered by the protein increased (i.e., as the protein expression level increased), a smaller percentage of the protein was used to form synapses.

For postsynaptic proteins, greater variability was observed. The negative correlation was found only in PSD95 (*p* = 0.005), where a larger area covered by this protein was associated with its smaller participation in synapses. In contrast, the percentage participation of Homer 1 in synapses was positively correlated (*p* < 0.0001) with the area occupied by this protein—the larger the area occupied by Homer 1, the greater its participation in synapses. No correlation was observed between the percentage participation of PICK1 and the number of immunopuncta (*p* = 0.78) or the area covered by the protein (*p* = 0.27). Detailed results are provided in the Supplementary Materials (Figure S4).

## 3. Discussion

This study is the first to our knowledge to provide a detailed analysis of both presynaptic and postsynaptic protein expression levels, as well as the excitatory synapse density throughout the day–night cycle under different conditions (LD 12:12 and LD 16:8), and in two constant conditions (DD and LL), alongside a thorough examination of locomotor activity in the fourth layer of the mouse somatosensory cortex. By identifying the endogenous basis of changes in synaptic protein levels and excitatory synapses, we explored how light influences synaptic plasticity in a circadian rhythm (Table 2 and Table 3).

### 3.1. Light Affects Locomotor Activity and Rhythm Robustness

When analyzing differences between animal activity phases (rest/activity), the consideration of single time points in each phase—one during the activity phase and one during the rest phase—appears to be sufficient, as demonstrated by Delorme et al. (2021) [44]. Since we had more time points per cycle, we performed an additional analysis comparing activity levels during the subjective day and night to better evaluate potential differences across various conditions. The largest discrepancies between subjective day–night and rest-activity phases were found under DD conditions. These differences are not surprising, given the gradual shift in the onset of locomotor activity toward the end of the subjective day under DD conditions, typical for free-running rhythm [45]. In constant darkness, animals lose the clear signal to initiate or end their locomotor activity, and their activity time is regulated exclusively by the circadian clock. However, the locomotor circadian activity can also be affected by sleep deprivation, hunger, and other stressors [46]. Although the daily shift in DD conditions was relatively small (on average, less than 20 min), our comparative analysis of periodograms revealed that the selected midpoint of the subjective night fell within a period of reduced, though still present, locomotor activity. Under DD conditions, rhythm stability was lower than in light–dark conditions (LD 12:12 and LD 16:8), reinforcing the idea that light not only maintains the timing of locomotor activity onset but also acts as a strong synchronizer of rhythmicity [47]. As Gonzalez (2018) noted, DD conditions can profoundly affect various parameters related to locomotor activity and rhythm desynchronization [48]. Furthermore, although DD seems to be an unstressful condition for nocturnal animals, rats exposed to constant darkness display depression-like behaviors [49].

Mice in the LD 16:8 conditions showed a notably shortened activity phase. Despite this, overall activity levels and activity distribution between the day and night under LD 16:8 conditions were comparable to LD 12:12, which aligns with findings from studies on Swiss Webster mice under similar conditions [50]. This is likely due to the synchronization of locomotor activity by light cues, although in the LD 16:8 conditions, the activity onset was delayed, occurring when the lights turned off.

Constant light disrupts the circadian clock, leading to the dysregulation of rhythms [24,25,26]. However, the duration of our experiment may not have been long enough to completely desynchronize the clock neurons [51]. It should also be noted that constant light is a very strong stress factor, especially for nocturnal animals [52,53]. Some mice in the LL conditions exhibited arrhythmicity, while others retained rhythmic locomotor activity, as observed by Ohta et al. (2005) [26]. Despite the change in many different parameters in LL conditions compared to LD 12:12, the activity levels during the subjective day and subjective night did not significantly differ from those during the rest and activity phases, respectively. Most rhythmic mice in the LL group, however, showed lower activity levels compared to other conditions, and rhythm stability was significantly reduced [54].

### 3.2. Presynaptic Protein Levels Are Clock-Dependent and Modulated by Light

Around 10% of transcripts in the cerebral cortex change their expression between day and night [55]. Additionally, 70% of mRNAs and 30% of phosphopeptides in synapses show rhythmic oscillations dependent on the time of day, highlighting the significant impact of the circadian clock on genes encoding synaptic proteins [56,57]. This study showed that in the mouse somatosensory cortex the circadian clock affected the increased presynaptic protein expression during the subjective day compared to the subjective night (Table 2).

Most presynaptic proteins, which are well-accepted synaptic markers, are present in both excitatory and inhibitory synapses [58]. In contrast, the postsynaptic proteins selected by us are associated with glutamate receptors and are predominantly found in excitatory synapses. Therefore, it is not surprising that a smaller percentage of presynaptic proteins was localized in excitatory synapses compared to postsynaptic proteins. This was particularly evident for NRXNs, which are also expressed in astrocytes [59]. Interestingly, we found that the increased expression of presynaptic proteins did not lead to a proportional increase in excitatory synapses, despite excitatory synapses making up more than 80% of synapses in the studied brain region [60].

Synaptophysin, a critical protein for synaptic vesicle formation and neurotransmitter release, plays a key role in synaptic plasticity [61]. It also forms complexes with synaptobrevin, which are involved in adjusting the circadian clock in response to light stimuli, allowing it to adapt to changes in the day–night cycle [62]. Although synaptophysin is crucial for resetting the clock [62], it did not show daily rhythmicity under LD 12:12 conditions in several brain regions, including the mouse cerebral cortex [8]. Our findings showed that the circadian clock drove an increase in synaptophysin 1 expression during the subjective day, a pattern that continued under light–dark conditions. Light amplified the day–night differences in synaptophysin 1 expression, though this effect diminished with prolonged light exposure. This discrepancy with the results of Sarowar et al. (2016) might result from the fact that they examined the entire cerebral cortex, without focusing on specific layers or regions, and such an approach could have masked region-specific changes [8]. It has been found that changes in synaptic protein composition and synapse number are species- and brain-region-specific, as well as influenced by experimental conditions [12].

Piccolo, a large protein crucial for the presynaptic active zone, regulates neurotransmitter release. It co-occurs with Bassoon, a more commonly used presynaptic marker, and both proteins share similar functions [58,63]. Piccolo helps organize active zones and anchor synaptic vesicles, ensuring efficient neurotransmitter release [63]. Additionally, Piccolo contributes to synaptic plasticity by regulating the ubiquitination of active zone proteins, maintaining synaptic stability [64,65].

Our research confirmed that Piccolo expression is regulated by the circadian clock [66]. In pinealocytes, where Piccolo is a part of the complex associated with synaptic ribbons, Piccolo levels are higher at night than during the day, with this pattern persisting in constant darkness [66]. In contrast, in the somatosensory cortex, we observed a cyclic increase in Piccolo expression during the subjective day and a decrease during the subjective night under DD conditions. Although this rhythm was partially masked in LD 12:12, under LD 16:8 conditions it was more pronounced, resembling the rhythm seen in DD. The changes in Piccolo expression under LL conditions were notable, as they deviated from the patterns observed in other conditions. We believe this can be explained by the locomotor activity patterns, as mice exhibited increased activity at the beginning of the subjective day only under LL. In all conditions, high locomotor activity coincided with lower Piccolo expression, suggesting that locomotor activity may play a key role in regulating Piccolo levels.

NRXNs, a family of cell adhesion proteins primarily found on presynaptic membranes, interact with neuroligins on the postsynaptic membrane [67]. Their interaction with neuroligins is important for synapse formation and stabilization, which is crucial for proper synaptic transmission [68,69]. NRXNs show circadian and daily expression patterns in the SCN, indicating circadian regulation, although not all variants follow the same rhythm pattern [7]. These rhythms in NRXNs expression may affect the excitatory-inhibitory balance in SCN synapses and contribute to daily synaptic remodeling [7]. Similarly, in our study, NRXNs were influenced by the circadian clock and modified by light under LD 12:12 conditions. Under prolonged light (LD 16:8), NRXN expression closely mirrored the pattern seen under LD 12:12 conditions, underscoring light’s role in modulating circadian changes.

Our data show that all analyzed presynaptic proteins are regulated by the circadian clock. Synaptophysin and Piccolo show minor modifications under LD 12:12, while long photoperiod exposure reveals a rhythmic pattern similar to LD 12:12. In LL conditions, Piccolo expression appears to be strongly connected with locomotor activity pattern.

### 3.3. The Clock-Driven Expression of Postsynaptic Proteins Is Linked to Locomotor Activity and Significantly Modified by Light

Similar to presynaptic proteins, the levels of postsynaptic proteins driven by the circadian clock increased during the subjective day. However, all postsynaptic proteins exhibited a decrease in expression at the beginning of the night, followed by an increase in the middle of the night.

PSD95, an important protein in excitatory synapses, is responsible for recruiting signaling components and maintaining synaptic structure and function [70]. We observed a reduction in PSD95 expression at the beginning of the subjective night in DD conditions when animal activity is the highest. Studies in other brain regions, including the SCN, hippocampus, and cerebral cortex, show that PSD95 levels increase at night under light–dark conditions [7,8,13], a trend that was also evident in our research. Although these changes occur in the dark phase, they appear to be light-dependent since locomotor activity at the start of the night did not significantly differ between LD 12:12 and DD. Long photoperiod and constant light exposure eliminate this rhythmicity entirely. Notably, PSD95 levels in LD 16:8 and LL conditions were consistently lower than in DD. It may indicate that light, as a strong stressor, not only shifted the increase in PSD95 to the night in LD 12:12 but also masked cyclic changes under excessive light exposure.

Homer proteins are crucial molecular adaptors that organize signaling components in the postsynaptic density and play a vital role in synaptic signaling and plasticity [71]. In the rat somatosensory cortex, Homer 1 mRNA levels rise at night (the active phase) compared to the day under LD 12:12 conditions [32]. Moreover, Homer 1a mRNA increases during wakefulness and decreases during sleep in several brain regions, especially after sleep deprivation [72,73]. In our study, we only observed changes in the distribution of Homer 1 under LD 12:12 conditions, what differs our finding and Nelson et al. (2004) report [32]. It is important to note that mRNA and protein levels can vary significantly across the circadian cycle, sometimes even showing opposing patterns [57]. In particular, Homer 1a protein is directed to the postsynaptic density during sleep, suggesting that while neuronal activity during wakefulness drives Homer 1a mRNA expression, it limits the transport of Homer 1a protein to the postsynaptic density [74].

Our findings showed that, under DD conditions, Homer 1 levels increased in the middle of the subjective night, when animal activity decreased. Interestingly, a second peak occurred in the middle of the day, just before the animals’ highest activity period, followed by a decline. Thus, the circadian clock influence on the Homer1 expression appeared to be negatively correlated with locomotor activity and was further modulated by light. Under LL conditions, Homer 1 also displayed bimodal patterns with peaks in the middle of both the subjective day and night, surprisingly mimicking the biological clock-driven changes seen under DD. Nevertheless, most circadian rhythms are disrupted under LL conditions [24,25,26], making it considerably more challenging to discern the mechanisms underlying Homer 1 oscillations in these conditions.

PICK1, a postsynaptic protein involved in the transport and regulation of AMPARs in excitatory synapses [75], in our study showed increased levels during the day in LD 12:12 conditions, similar to the pattern seen in DD during the middle of the subjective night and beginning of the subjective day. Comparing the locomotor activity of animals in both conditions, the increase in PICK1 appeared more related to decreased or absent activity rather than light exposure. The earlier rise in PICK1 under constant darkness could be linked to the earlier onset of locomotor activity in DD, which shifts the rest phase. Under long photoperiod or constant light, all rhythmic changes in PICK1 expression were abolished.

Thus, our findings showed that the circadian clock regulated postsynaptic protein expression, which was also modulated by light. Additionally, a stronger relationship was observed between locomotor activity and postsynaptic proteins compared to presynaptic ones.

### 3.4. Light Modifies Endogenous Changes in Excitatory Synapses

The use of double-immunohistochemical labeling for selected presynaptic and postsynaptic protein pairs allows for the precise identification of excitatory synapses in accordance with the method widely employed in studies of synaptic plasticity, neurodegeneration, and mechanisms of synaptic transmission [76,77,78,79,80,81]. Although presynaptic markers are also present in inhibitory synapses [58], their colocalization with postsynaptic markers specific to excitatory synapses confirms the accurate identification of excitatory synapses and proper assessment of their circadian changes. Under LD 12:12 conditions, we observed consistent synapse density values across different protein pairs, ensuring reliability of the results. The lower excitatory synapse density in this study (using immunohistochemistry) compared to our previous reports (using TEM) [30] is probably attributed to methodological differences [58].

In DD conditions, the pattern of circadian excitatory synapse density remained stable, regardless of the synaptic markers used. Synapse density increased during the middle of the subjective night, stayed elevated at the start of the subjective day, and then decreased, reaching a minimum at the beginning of the subjective night. Our previous studies did not detect differences in excitatory synapse density between the subjective day and subjective night in DD conditions [30], probably due to the analysis being limited to only two time points. In the present, extended study, we identified circadian fluctuations in excitatory synapse density in DD conditions, which were not identified before.

The lowest excitatory synapse density at the start of the subjective night coincided with the highest level of locomotor activity in DD conditions (see Figure 2 and Figure 5). The subsequent increase in synapse density in the middle of the subjective night aligned with the decline in locomotor activity. These findings are consistent with observations under LD 12:12 conditions, where increased nighttime locomotor activity correlates with decreased excitatory synapse density in the somatosensory cortex [30]. Our results confirmed earlier predictions that the observed asymmetry in excitatory synapse rhythm could be driven by the circadian clock and locomotor activity [30]. The hypothesis that the decrease in excitatory synapses is related to the “anticipation” of light is not supported by the current results under DD conditions. Instead, excitatory synapse density appears to be regulated by the circadian clock, with a clear impact of the locomotor activity [30].

Our current study revealed that this pattern also existed in LD 12:12 conditions, with synapse density rising during the day and falling at night. Interestingly, it mirrored the changes observed in DD conditions, although there was a shift, similar to the shift in locomotor activity, between LD 12:12 and DD conditions.

In LD 16:8 conditions, we observed a similar trend, with excitatory synapse density peaking at the start of the day and reaching a minimum at the beginning of the night, though this was most pronounced in Pic+/Hom+ synapses. Light exerted a stronger influence on excitatory synapse density under LD16:8, masking the endogenous fluctuations. Despite the alterations in certain synaptic proteins observed in LL conditions, no significant changes in excitatory synapse density were detected, which remained stable, regardless of the synaptic markers used.

### 3.5. Postsynaptic Markers Reveal Distinct Roles of Glutamate Receptors in Circadian Synaptic Plasticity

The selection of three distinct postsynaptic markers in this study allowed for initial insights into potential changes linked to different glutamate receptors. However, determining the association with specific receptor types was challenging because all these postsynaptic proteins are indirectly involved in modulating synaptic plasticity across multiple receptor types [82,83,84,85]. In excitatory synapses of the cerebral cortex, various glutamatergic receptors work in concert to regulate signaling, synaptic plasticity, and neuronal adaptation, processes critical for brain function [86,87,88].

PSD95 plays a significant role in stabilizing and regulating N-Methyl-D-Aspartate (NMDA) receptors in the postsynaptic membrane [89]. Although PSD95 does not directly bind to AMPA receptors (AMPARs) [85], it influences their regulation through interactions with stargazin, which delivers AMPARs to the synaptic membrane [85,90,91]. PSD95 also plays a role in “silent synapses”, incorporating AMPARs during long-term potentiation (LTP) via NMDA receptor activation [82,92].

Homer proteins directly interact with group I metabotropic glutamate receptors (mGluRs), primarily mGluR1 and mGluR5, supporting calcium signaling and promoting protein complex formation at synapses [93]. Homer 1a, in particular, binds to mGluR5 in the postsynaptic density and plays a role in AMPAR removal from synapses following activation by neuromodulators [74,94].

PICK1, another postsynaptic protein, is essential for AMPAR trafficking, facilitating their transport from the endoplasmic reticulum to the postsynaptic membrane [75] and promoting AMPAR endocytosis and recycling [95]. Changes in PICK1 levels can significantly influence synaptic strength by modulating the number of AMPARs at the postsynaptic membrane [96].

Under DD conditions, we found more Syp1+/PSD95+ synapses compared to Pic+/Homer+ synapses, with more pronounced circadian changes in Syp1+/PSD95+ and NRXN+/PICK1+ synapses than in Pic+/Hom+ synapses. This suggests that ionotropic receptors play a more prominent role than metabotropic receptors in constant darkness.

Under LD 12:12 conditions, an increase in mGluR5 receptor levels was observed in various brain regions during the day [97], which aligns with the rise in Pic+/Homer+ synapse density observed in our studies. The consistent pattern of Pic+/Homer+ synapses in both LD 12:12 and DD conditions suggests that the circadian clock regulates the rhythmicity of excitatory synapses containing metabotropic receptors, independent of locomotor activity. We also observed a marked increase in Syp1+/PSD95+ synapses during the day and a decrease at night under LD 12:12 conditions. This is consistent with findings from the CA1 region of the hippocampus, where NMDA receptor activity decreases during the dark phase [98,99]. However, our results differ from those in the lateral hypothalamus, where VGluT2+/PSD95+ synapses increase at night [100]. These regional differences underscore the importance of context when interpreting findings specific to different brain areas [12,101]. NRXN+/PICK1+ synapses exhibited the smallest circadian changes, though their numbers dropped sharply in the middle of the night, in line with the previous research under LD 12:12 conditions [30].

In contrast, under LD 16:8 conditions, circadian changes in synaptic density were less noticeable than in LD 12:12 and DD conditions, and no circadian oscillations were detected in LL conditions. However, we found a higher number of Pic+/Homer+ synapses compared to the other synapses in both conditions. This may suggest that metabotropic receptors are more actively involved in adapting to excessive light exposure.

PSD95 also contributes to dendritic spine maturation [102], which is characterized by the formation of mushroom-shaped spines containing a spine apparatus [103,104]. In the fourth layer of the mouse somatosensory cortex, a higher number of mature, mushroom-shaped spines with spine apparatuses are observed during the day compared to the night under LD 12:12 conditions, suggesting that light promotes dendritic spine maturation [105]. Interestingly, while these results correlate with the higher density of Syp1+/PSD95+ synapses observed during the day under LD 12:12 conditions, they do not correspond with changes in PSD95 expression levels, confirming an independent regulation of synaptic density and PSD95 expression [106,107].

Since mushroom spines are enriched with AMPARs compared to the other spines [108,109,110], it is interesting to consider to what extent PICK1, which traffics AMPARs, may contribute to this process. Despite an increase in PICK1 protein expression during the day, NRXN+/PICK1+ synapses remained stable at the start of the night, though they dropped significantly by midnight. This finding highlights the complex role of PICK1 in AMPAR transport, driven by the bidirectional process of its trafficking [75,95]. In DD conditions, the changes in PICK1 expression and NRXN+/PICK1+ synapse density followed the same pattern, indicating that light influences the number of these synapses in LD 12:12 conditions. The results of studies vary depending on the brain region and AMPAR subunit [111,112]. In the SCN, GluR2/3-containing AMPARs decrease during the subjective night under DD conditions [111], while in the cortex and hippocampus, GluR1-containing AMPARs increase during wakefulness [112].

### 3.6. Functional Implications and Possible Mechanisms of Circadian Changes in Excitatory Transmission

Circadian changes in excitatory transmission are essential for the functioning of the nervous system, especially in the context of adaptation to changing environmental conditions. In constant darkness, we observed a decrease in the density of excitatory synapses at the beginning of the subjective night, which correlated with an increase in the animals’ locomotor activity. Our observations are consistent with the theory of homeostatic synaptic plasticity [113,114], which suggests that there are adaptive processes regulating neuronal activity at the synaptic level. A decrease in excitatory synapses in the somatosensory cortex during periods of intense activity could serve a compensatory function, reducing excessive sensory input associated with whisker use. As a result, there might be a greater selection of stimuli, thereby increasing the precision of critical information processing.

According to the homeostatic synaptic plasticity hypothesis, the increase in the number of excitatory synapse density during the day (light phase) that we observed under LD 12:12 conditions could result in enhanced sensory sensitivity [113,114], enabling nocturnal animals to react more quickly to potential threats. However, this increase may not directly indicate greater synaptic transmission but rather an adaptive “preparation” for potential dangers. The above mechanisms may have evolved as adaptive strategies to improve responsiveness to environmental changes, thereby enhancing the survival of nocturnal animals.

In LD 12:12 conditions, glucocorticoid levels in nocturnal animals increase at the beginning of their active phase, i.e., at night [115,116]. Studies by Ishida et al. (2005) have shown that increased exposure to light, especially during the active phase of animals, leads to a growth in corticosterone levels in the plasma and brain despite the lack of activation of the hypothalamic–pituitary–adrenal axis [117]. Constant light, which is an exceptionally strong stressor, especially for nocturnal animals, resulted in the cessation of rhythmic synaptic changes, which is consistent with the results obtained in other brain regions [20,29]. Liston et al. (2013) demonstrated that chronic and excessive glucocorticoid exposure impaired memory by eliminating newly formed learning spines, and in the study of Schröder et al. (2023), constant light impaired spatial working memory in the hippocampus [20,118]. In our study, the number of excitatory synapses remained at an elevated level throughout the subjective day–night cycle, and it appears to be the result of increased alertness, but the lack of rhythmic changes suggests that this might happen at the expense of cognitive functions such as memory and learning.

### 3.7. Limitations of the Study and Future Directions

Our research is focused on a specific region of the somatosensory cortex, and it is well established that synapse density varies significantly across different brain regions. While the double-immunofluorescence technique combined with confocal scanning microscopy enabled us to analyze synapse density, it is important to note that this is an ex vivo study, meaning we cannot definitively assess the functional activity of the synapses analyzed. Additionally, although confocal microscopy allows for the examination of larger tissue areas this comes at the cost of lower resolution compared to transmission electron microscopy (TEM). This study focuses on the short-term effects of varying lighting conditions on synaptic proteins and synapse density without assessing the potential long-term impacts. Future research could explore the chronic effects of continuous light or darkness exposure to better understand the sustained influences of these conditions on brain function and structural plasticity.

## 4. Materials and Methods

### 4.1. Animals

The study was designed and conducted on 128 male C57BL/6 mice aged five to six weeks (Experimental Medicine Centre at the Medical University of Bialystok, strain imported from The Jackson Laboratory, Bar Harbor, ME, USA), adhering to the Council Directive 2010/63EU of the European Parliament and the Council of 22 September 2010 on protecting animals for scientific purposes. The study was approved by the Animal Care and Use Committees of the Jagiellonian University in Krakow, Poland.

### 4.2. Locomotor Activity Under Different Lighting Conditions

All animals were habituated for two weeks under light–dark conditions (12 h of light and 12 h of darkness; light 60 lx) at 25 °C and 50% humidity in a soundproof-insulated locomotor activity recording room. From the beginning of experiments, each mouse was individually housed in a cage fitted with a running wheel coupled to a 16-channel electromagnetic pulse counter (MIKI 1; Autel, Krakow, Poland) to precisely capture movement data [105,119]. After habituation, mice were divided into four groups: LD 12:12 group (n = 32), DD group (n = 32), LD 16:8 group (n = 32), and LL group (n = 32). For the next 10–14 days, they were kept under the same temperature, humidity and in the respective light regime: LD 12:12 group under 12 h of light and 12 h of darkness conditions, LD 16:8 group under 16 h of light and 8 h of darkness conditions, DD group under constant darkness; and LL group under constant light. The animals were fed a standard diet and water ad libitum.

The locomotor activity of mice was monitored continuously throughout the experiments. Next, the obtained data were analyzed using the NIH ImageJ 1.49m software with the ActogramJ plugin, designed for detailed analysis of circadian rhythms (http://imagej.nih.gov/ij; accessed on 30 January 2018).

As revealed by actograms (Figure 2A–D), all mice from the LD 12:12, LD 16:8, and DD groups showed locomotor rhythmicity. In contrast, approximately half of the LL mice had some degree of arrhythmicity, or their locomotor rhythmicity was maintained but stayed low. The remaining mice in the LL group were rhythmic. Because arrhythmia or low activity intensity is not unusual under LL conditions [120], all mice were selected for subsequent experiments.

Mice from each group were killed at 6-h intervals (Figure 2E–H) during the 24-h cycle at ZT0/CT0, ZT6/CT6, ZT12/CT12, and ZT18/CT18. Here, ZT refers to Zeitgeber time, which is the time of day in relation to the light–dark cycle, and CT refers to circadian time, which is the time of day in constant conditions designed to detect an input from the circadian clock. ZT0/CT0 marks the beginning of the day/subjective day, and ZT12/CT12 marks the beginning of the night/subjective night. Each ZT/CT subgroup consisted of 8 mice.

#### Parameters Related to Daily/Circadian Rhythmicity

Our previous studies showed that the overall activity level and the period of locomotor activity in animals kept under 12 h light:12 h dark, and constant darkness conditions are similar [30]. In this study, we performed a more complex analysis of several additional parameters and using additional light conditions, which correspond to different activity patterns characterizing mice kept under long photoperiod and constant light conditions. The analysis was performed separately in each group of animals for at least eight consecutive days, starting no earlier than 24 h after the change in light conditions (excluding the day when the brains were collected) using the ActogramJ plugin for ImageJ.

The following parameters, described previously by other authors, were analyzed: tau, delta, alpha, rho, and overall activity [44,121,122].

Tau, the period of the daily/circadian rhythm, was calculated using periodograms on ImageJ with the ActogramJ plugin, choosing the Fourier index for its highest reliability [123] and using the software https://circadian.org/periodogram.html (accessed on 30 July 2023) [124].

Alpha and delta were calculated based on actograms using the ImageJ program with the ActogramJ plugin. Alpha denotes the period of sustained activity, i.e., the number of hours between the onset and offset of activity. Delta denotes the sum of all shifts in the activity onset over the following eight day–night (or subjective day–night) cycles. In the LL group, these parameters could not always be calculated due to weak rhythmicity or arrhythmicity in some animals [120]. Rho determines the length of the rest period and was calculated based on the previously established parameters—tau and alpha.

The overall activity was calculated as the average number of wheel revolutions during a single day–night or subjective day–night cycle.

We also calculated activity levels in the activity and rest phases based on the collected data. In the LD 12:12 group, the locomotor activity of animals during the activity phase was equivalent to their activity at night. In contrast, their activity during the rest phase was equivalent to that during the day. To calculate the activity of animals in the activity and rest phases in the DD, LD 16:8, and LL groups, the entire cycle period (tau) was divided into two halves. One of these halves represented the rest phase and the other the activity phase, with the beginning of the activity phase marking the beginning of the animal’s highest activity [44], as shown in Figure S1.

Under constant conditions (DD and LL), rho and alpha do not reflect the length of subjective day and night. To freely compare the time points of 6-h intervals with the analogous time points in LD 12:12 conditions, an additional analysis of activity during subjective day and subjective night was performed. In the groups of animals remaining under constant light conditions (DD and LL conditions), the subjective day–night cycle was divided based on the acclimatization period in light–dark conditions into subjective day (light on in light–dark conditions during acclimatization; CT0–CT12) and subjective night (light off in light–dark conditions during acclimatization; CT12–CT0). On this basis, subjective night and subjective day activities were calculated in the DD and LL groups.

Additionally, the percentage of daily activity compared to total activity, % day/subjective day, i.e., percentage of daily activity/subjective day compared to total activity, was calculated.

The Qp index, which determines the robustness of daily/circadian rhythm, was calculated using the Circadian.org/periodogram.html program [124], with a higher Qp value indicating greater rhythm solidity. The Qp coefficient was normalized and rescaled to obtain %Qp according to Pfeffer (2017) [125].

### 4.3. Immunohistochemistry Procedure

#### 4.3.1. Fixation and Sectioning

The mice were deeply anesthetized with Morbital (100 mg/kg b.w.; Biowet, Puławy, Poland) and intracardially perfused with saline solution followed by 100–150 mL of fixative buffer (4% paraformaldehyde in 0.1 M phosphate buffer in 0.1 M phosphate buffer; pH 7.4). The brains were removed immediately after perfusion and left in 0.1 M PBS at 4 °C.

Next day, slices of 30 µm in thickness were cut tangentially to the surface using a vibratome (Leica VT1000S, Leica Biosystems Nussloch GmbH, Nussloch, Germany). Sections were examined under a stereomicroscope (Optiphot Nikon, Tokyo, Japan), and those containing the barrel field cortex were selected and mounted on polylysine-coated microscope slides (Polysine Menzel-Glaser, Thermo Scientific, Waltham, MA, USA). The slides with brain sections were then stored at 4 °C. The sections were photographed (Optiphot Nikon, Japan) and the stacks of images were used to select only those containing row B of the barrel cortex [126].

#### 4.3.2. Double-Immunofluorescence Staining and Confocal Laser Scanning Microscopy

A double-immunohistochemical staining protocol using antibodies originating in the same species was adapted from Negoescu et al., 1994; Brouns et al., 2002; Johnson and Spence (eds.), 2010 (Section 6.2) [127,128,129].

First staining—immunofluorescence detection of presynaptic proteins. The sections were consecutively treated with streptavidin (for 15 min; Invitrogen, Carlsbad, CA, USA; 434301), biotin (for 15 min; Invitrogen, Carlsbad, CA, USA; B1595), and H_2_O_2_ (0.3% H_2_O_2_ in 0.1 M PBS for 15 min) to block binding with endogenous constituents and washed after every step in 0.1 M PBS. For permeabilization of cell membranes, the sections were treated with 0.1 M PBS containing 0.5% Triton X-100 for 1 h. Next, the sections were incubated for 1 h in a blocking solution of 5% normal goat serum in 0.1 M PBS and then for 30–60 min in 1% bovine serum albumin (BSA; Thermo Scientific, Waltham, MA, USA) to quench non-specific binding.

The sections were incubated with the first primary antibody against presynaptic proteins: synaptophysin 1 (1:2000; Synaptic Systems GmbH, Goettingen, Germany, #101 002), Piccolo (1:1000; Synaptic Systems GmbH, Goettingen, Germany, #142 002) or neurexin 1/2/3 (1:500; Synaptic Systems GmbH, Goettingen, Germany, #175 003) diluted in 1% BSA overnight at 4 °C. Next day, the sections were washed for 3 × 5 min in 0.1 M PBS and detection of the primary antibody was performed with biotin-conjugated affinity-purified goat anti-rabbit immunoglobulin IgG (H + L; Jackson Immunoresearch Europe Ltd., Ely, UK; 111-065-144) in 1:1000 dilution in 1% BSA for 1 h and horseradish peroxidase conjugated with streptavidin (Thermo Scientific, Waltham, MA, USA) diluted 1:100 in 1% BSA for 1 h and washed for 3 × 5 min in 0.1 M PBS between steps. Visualization was performed using Alexa 488 conjugated tyramide diluted 1:100 in amplification buffer (Invitrogen, Carlsbad, CA, USA; T20948) for 10 min followed by washing for 3 × 2 min in 0.1 M PBS.

Second staining—immunofluorescence detection of postsynaptic proteins. Second labeling required re-blocking non-specific binding of biotin, streptavidin, and horseradish peroxidase. The presynaptic proteins-labeled sections were incubated in streptavidin (for 15 min; Invitrogen, Carlsbad, CA, USA), biotin (for 15 min; Invitrogen, Carlsbad, CA, USA), and H_2_O_2_ (0.3% H_2_O_2_ in 0.1 M PBS for 15 min), and washed in 0.1 M PBS between subsequent steps. Next, the sections were treated with Fab fragments of affinity-purified goat anti-rabbit immunoglobulin IgG (H + L; Jackson Immunoresearch Europe Ltd., Ely, UK; 111-067-003) diluted 1:100 for 3 h to remove free binding sites of the anti-rabbit immunoglobulin and rinsed with 0.1 M PBS for 3 × 5 min. Then, the synaptophysin 1-labeled sections were incubated with the second primary antibody against postsynaptic proteins PSD95 (1:2000; Abcam, Cambridge, UK, ab18258), Piccolo-labeled sections with antibody against Homer 1 (1:2000; Synaptic Systems GmbH, Goettingen, Germany, #160 003), and neurexin 1/2/3-labeled sections with antibody against PICK1 (1:500; Abcam, Cambridge, UK, ab3420) diluted in 1% BSA overnight at 4 °C. The next day, the sections were washed for 2 × 2 min in 0.1 M PBS, and primary antibody detection was performed with biotin-conjugated affinity-purified goat anti-rabbit immunoglobulin IgG (H + L; Jackson ImmunoResearch, West Grove, PA, USA, 111-065-144) diluted 1:1000 in 1% BSA for 1 h and streptavidin-HRP diluted 1:100 in 1% BSA for 1 h and washed for 2 × 2 min in 0.1 M PBS between subsequent steps. Visualization was performed using Alexa 647 conjugated tyramide diluted 1:100 in amplification buffer (Invitrogen, Carlsbad, CA, USA, T20951) for 10 min followed by washing for 2 × 2 min in 0.1 M PBS. DAPI was used to stain the cell nuclei. Negative control was prepared by omitting the primary antibodies.

#### 4.3.3. Image Acquiring

The labeled tissue was examined with confocal laser scanning microscope fitted with inverted microscope support equipped with 405 nm, 473 nm, and 635 diode lasers (Olympus Fluoview FV1200, Tokyo, Japan). Fluorescence images were acquired sequentially under magnification 60× oil objective (Plan-Apochromat NA 1.42; Olympus, Tokyo, Japan) and 4.5× digital zoom, with a linear scan speed of 12.5 µm/pixel and step size of 0.46 µm on the Z axis. Detector gain voltages and pinhole were set at the beginning of the experiment and maintained constant during the acquisition of all images [130,131,132]. Row B of the barrel cortex was identified by its clearly visible nuclei pattern and was selected for further analysis. Four to six optical sections showing labeling of both synaptic markers were selected per stack, and two or three stacks per section, generating an average of eight to twelve images.

#### 4.3.4. Quantitative Analysis of Synaptic Protein Expression and Colocalization Analysis

All measurements were performed using NIH ImageJ 1.49m software (http://imagej.nih.gov/ij; accessed on 30 January 2018). The images were split into separate channels, and only red and green channels were selected for further analysis. The preparation of images before measurements included background subtraction and noise removal (median and watershed filters) [133]. The level of protein expression was determined by two parameters: an area covered by protein clusters (area fraction; Analyze Particles plugin) and a number of discrete puncta (immunopuncta; Analyze Particles plugin). Positive correlations were found between both parameters for all synaptic proteins (Supplementary Materials, Figure S2). To count synapses, we considered the co-labeled points indicating colocalization of presynaptic and postsynaptic proteins from each pair, which we obtained using the AND function of the ImageJ calculator. Next, the number of immunopuncta was converted to synapse density, considering the image area and the optical section thickness.

### 4.4. Statistical Analysis

All data are given as mean ± SEM. To compare locomotor activity parameters between animal groups and to analyze other parameters across specific time points within groups (including the number of protein immunopuncta, area covered by proteins, synapse density, and percentage content of synaptic proteins), a one-way analysis of variance (ANOVA) was performed. Tukey’s post hoc test was used to identify specific group pairs with differences. Alternatively, if data were not normally distributed or variances differed significantly, the Kruskal–Wallis test with Dunn’s post hoc test was applied.

The Pearson correlation (or the Spearman correlation for not normally distributed data) was used to test relationships between the duration of the active phase and break, the number of immunopuncta and area covered by protein, as well as the percentage content of protein in synapses with both the number of immunopuncta and the area covered by protein. All data were analyzed using GraphPad Prism 5.01 software (GraphPad Software Inc., La Jolla, CA, USA).

## 5. Conclusions

The present study demonstrated that presynaptic and postsynaptic proteins in the mouse somatosensory cortex exhibit circadian expression changes, which are modulated by light. Locomotor activity seems to be more strongly linked to postsynaptic proteins than to presynaptic ones. Notably, excitatory synapse density is regulated by the circadian clock and inversely correlated with locomotor activity. Excessive light exposure disrupts or even abolishes cyclical synaptic changes, but it sustains an elevated level of excitatory synapses throughout the subjective day–night cycle.

## Figures and Tables

**Table 1:** Analyzed locomotor activity parameters.

Parameter	Definition
*Tau*	The period of the daily (day–night) or circadian (subjective day–subjective night) rhythm.
*Delta*	The shift (phase advance or phase delay) of the activity onset.
*Alpha*	The duration of the activity phase; the time between the onset and offset of activity.
*Rho*	The duration of the rest phase; determined by the total cycle period (*tau*) and the activity phase (*alpha*).
Overall activity	The total number of wheel revolutions during one day–night or subjective day–subjective night cycle.
Qp	The robustness of the daily (day–night) or circadian (subjective day–subjective night) rhythm; an indication of the rhythm’s stability.
Night/subjective night activity	The number of wheel revolutions during the night (ZT12-ZT24) or subjective night (CT12-CT24).
Day/subjective day activity	The number of wheel revolutions during the day (ZT0-ZT12) or subjective day (CT0-CT12).
Activity phase	The number of wheel revolutions during the activity phase (*alpha*).
Rest phase	The number of wheel revolutions during the rest phase (*rho*).

ZT—Zeitgeber time; CT—circadian time.

**Table 2:** Changes in the expression levels of presynaptic and postsynaptic proteins influenced by examined factors.

Protein		Endogenous Effect (DD Conditions)	Effect of Light (12 h Light)	Effect of Prolonged Light (16 h Light)	Effect of Constant Light (24 h Light)
**Presynaptic proteins**	Syp1	increase during the day ↑CT0	enhancing the cyclic changes ↑ZT6	maintaining the cyclic changes ↑ZT0	masking cyclic changes
	Pic	increase during the day ↑CT0, ↑CT6	maintaining the cyclic changes ↑ZT6	maintaining the cyclic changes ↑ZT0, ↑ZT6	decrease during the day ↓CT0 *
	NRXN	increase during the day ↑CT6	decrease during the day ↓ZT6	decrease during the day ↓ZT6	masking cyclic changes
**Postsynaptic proteins**	PSD95	decrease at night ↓CT12	increase at night ↑ZT12, ↑ZT18	masking cyclic changes	masking cyclic changes
	Hom1	increase in the middle of the day and the night ↑CT6, ↑CT18	masking cyclic changes	maintaining cyclic changes at night and an increase at the beginning of the day ↑ZT0, ↑ZT18	maintaining cyclic changes ↑CT6, ↑CT18 *
	PICK1	increase at the beginning of the day and in the middle of the night ↑CT0, ↑CT18	maintaining during the day and masking cyclic changes at night ↑ZT0, ↑ZT6 *	masking cyclic changes	masking cyclic changes

* Conditions where there is a clear connection between locomotor activity and the observed changes. The terms “day” and “night” refer to the subjective day and subjective night under constant conditions (DD and LL). Specific time points are included for clarity: ZT0/CT0 represents the beginning of the day/subjective day, ZT6/CT6 the middle of the day/subjective day, ZT12/CT12 the beginning of the night/subjective night, and ZT18/CT18 the middle of the night/subjective night. Syp1, synaptophysin 1; Pic, Piccolo; NRXNs, neurexin 1/2/3; Hom1, Homer 1. ↑ indicates an increase at the time point, while ↓ indicates a decrease at the time point.

**Table 3:** Changes in the density of excitatory synapses influenced by analyzed factors.

Synapse	Endogenous Effect (DD Conditions)	Effect of Light (12 h Light)	Effect of Prolonged Light (16 h Light)	Effect of Constant Light (24 h Light)
Syp1+/PSD95+	increase during the day, and decrease at night ↑CT0, ↓CT12	increase during the day ↑ZT0, ↑ZT6	increase during the day ↑ZT0	masking endogenic changes
Pic+/Hom1+	decrease at night ↓CT12	increase during the day ↑ZT0, ↑ZT6	decrease at ZT12 ↓ZT12	masking endogenic changes
NRXN+/PICK1+	decrease followed by an increase at night ↓CT12 ↑CT18	decrease at night ↓ZT18	masking endogenic changes	masking endogenic changes
Summary of excitatory synapses	decrease in the night ↓CT12	increase in the day and decrease at night ↑ZT0, ↑ZT6, ↓ZT18	masking endogenic changes	masking endogenic changes

The terms “day” and “night” refer to the subjective day and subjective night under constant conditions (DD and LL). Specific time points are included for clarity: ZT0/CT0 represents the beginning of the day/subjective day, ZT6/CT6—the middle of the day/subjective day, ZT12/CT12—the beginning of the night/subjective night, and ZT18/CT18—the middle of the night/subjective night. This table includes data on synapses visualized by co-labeling the following proteins: Syp1+/PSD95+ (Synaptophysin 1—PSD95), Pic+/Hom1+ (Piccolo—Homer 1), and NRXN+/PICK1+ (Neurexin 1/2/3—PICK1). ↑ indicates an increase at the time point, while ↓ indicates a decrease at the time point.

## Data Availability

All relevant data are included in the paper and Supplementary Materials. Further inquiries can be directed to the corresponding author.

## Supplementary Materials

The following supporting information can be downloaded at: https://www.mdpi.com/article/10.3390/ijms252312870/s1.

## Abbreviations

DD, constant darkness; LD 12:12, light–dark 12 h light: 12 h darkness; LD 16:8, light–dark 16 h light: 8 h darkness; LL, constant light; ZT, Zeitgeber time; CT, circadian time; TEM, transmission electron microscopy; AMPAR, alpha-amino-3-hydroxy-5-methyl-4-isooxazole-propionic acid receptor; mGluR, metabotropic glutamate receptor; Arc, activity-regulated cytoskeleton-associated protein; NMDAR, N-Methyl-D-Aspartate receptors; Syp1, synaptophysin 1; NRXNs, neurexin 1/2/3; PSD95, postsynaptic density protein 95; PICK1, protein interacting with C alpha kinase 1; tau, period of biological rhythm; delta, shift of the activity onset; alpha, duration of activity phase; rho, duration of rest phase.
