# Supplementary material for: Light-Modulated Circadian Synaptic Plasticity in the Somatosensory Cortex: Link to Locomotor Activity
Source: Int J Mol Sci. 2024 Nov 29;25(23):12870. doi: 10.3390/ijms252312870 (PMC11641775; doi:10.3390/ijms252312870)
Supplement: Supplementary file 1 [file ijms-25-12870-s001.zip › ijms-3312893-supplementary.pdf]

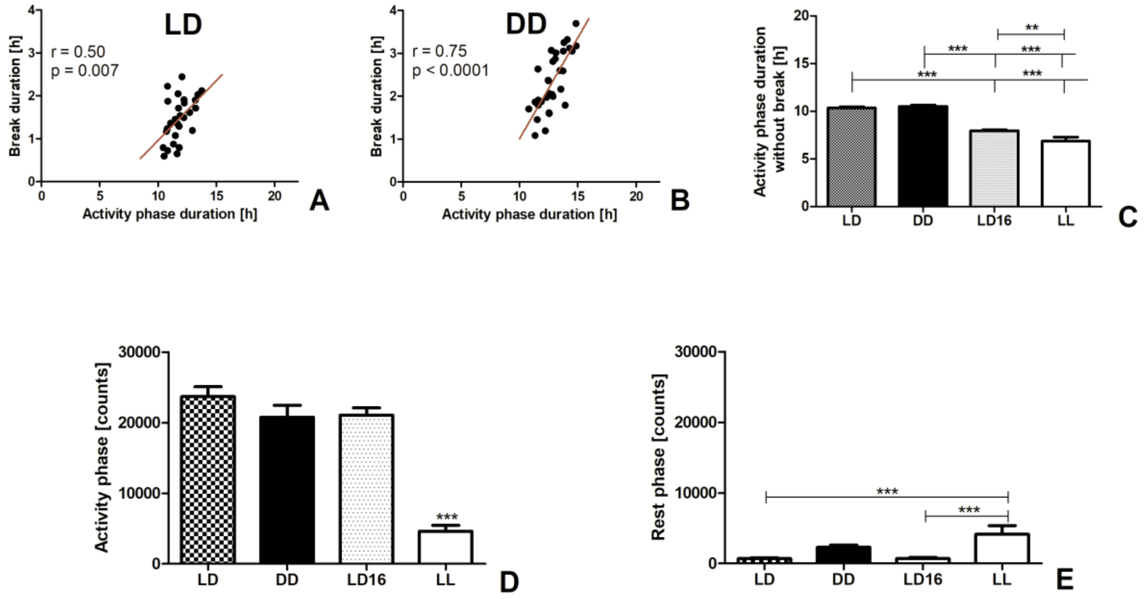

Figure S1: Analysis of daily and circadian rhythmicity. (A-B) Correlation between the length of breaks during the animals' high activity period and the duration of the activity phase (alpha) under different conditions. r, Pearson correlation coefficient (C) Activity phase duration (alpha) without breaks. Locomotor activity levels during (D) the rest and (E) activity phases under different conditions. The graphs show means ± SEM (one-way ANOVA; *** $p < 0.001$). The experimental light conditions: light-dark 12h:12h (LD), constant darkness (DD), prolonged light LD 16h:8h (LD16), and constant light (LL).

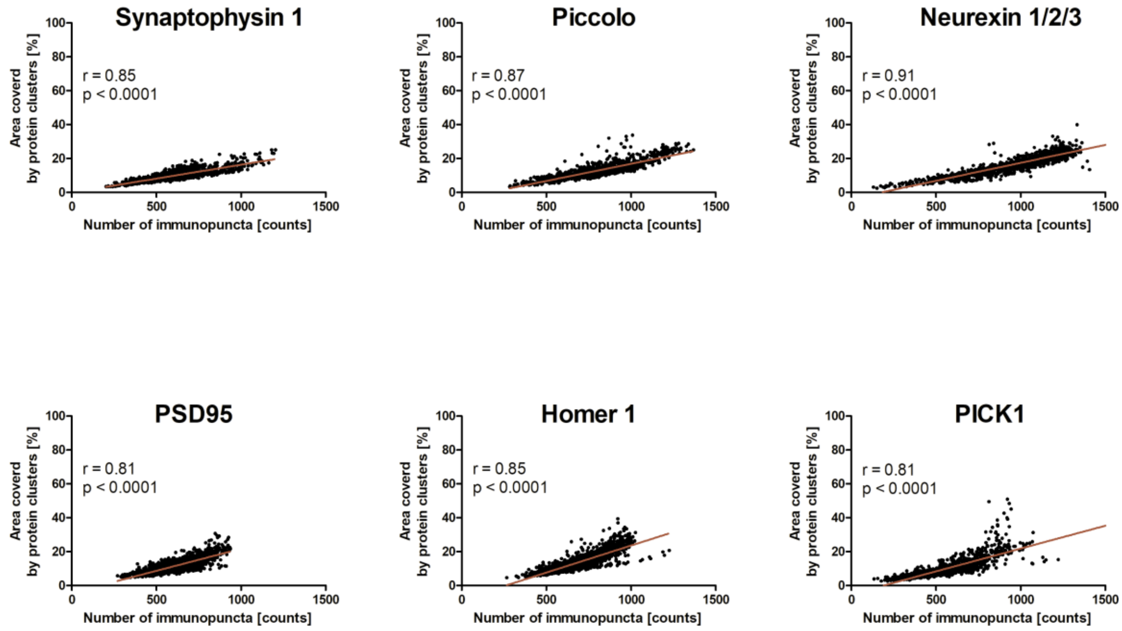

Figure S2: Correlation between the number of protein immunopuncta and the area covered by that protein for respective presynaptic (top panel) and postsynaptic protein (bottom panel). r, Pearson correlation coefficient.

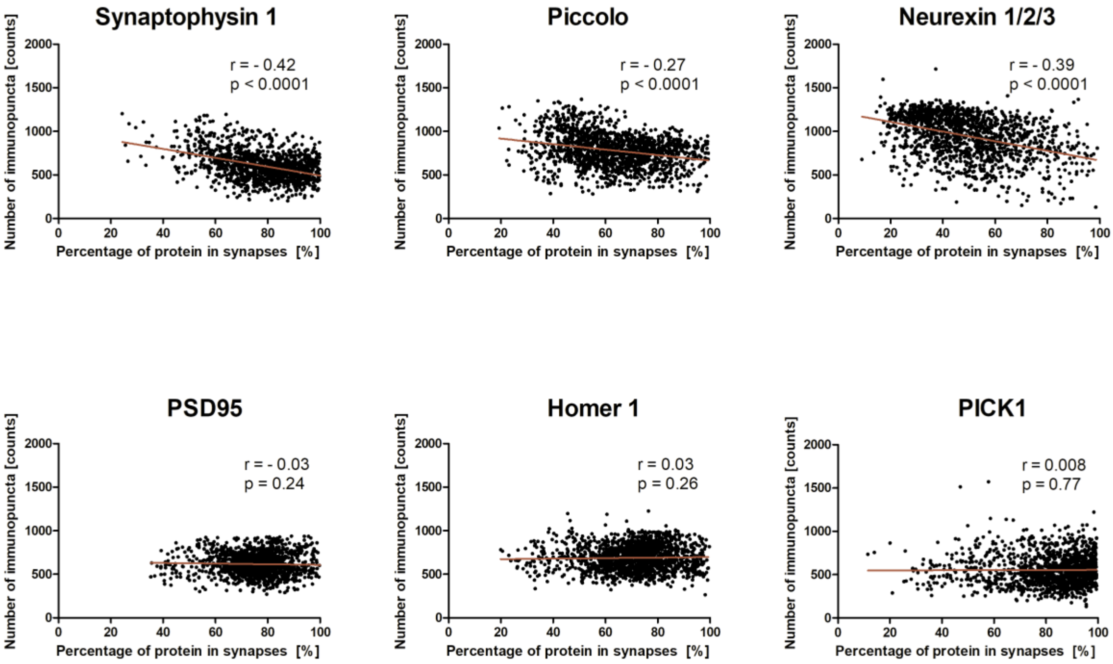

Figure S3: Correlation between the number of protein immunopuncta and the percentage participation of that protein in excitatory synapses for presynaptic proteins (top panel): (A) Synaptophysin 1, (B) Piccolo, (C) Neurexin 1/2/3 and postsynaptic proteins (bottom panel): (D) PSD95, (E) Homer 1, (F) PICK1. r, Pearson correlation coefficient.

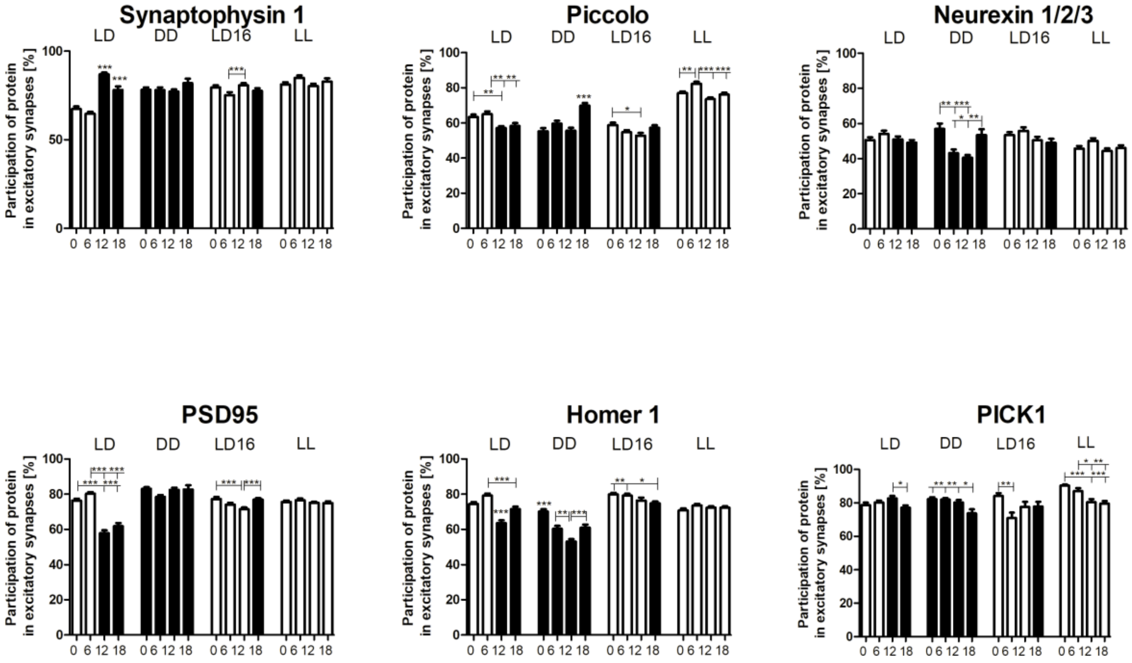

Figure S4: Daily and circadian changes in the percentage content of respective presynaptic (top panel) and postsynaptic proteins (bottom panel) throughout the day/night or the subjective day/night cycle under different lighting conditions. The graphs show means ± SEM (one-way ANOVA; *** $p < 0.001$, ** $p < 0.01$, * $p < 0.05$). The asterisks located directly above the bars signify that the difference applies to all time points within the group. The experimental light conditions: light-dark 12h:12h (LD), constant darkness (DD), prolonged light LD 16h:8h (LD16), and constant light (LL). Time points are defined as follows: 0 – ZT0/CT0: the beginning of the day/subjective day; 6 – ZT6/CT6: the middle of the day/subjective day; 12 – ZT12/CT12: the beginning of the night/subjective night; 18 – ZT18/CT18: the middle of the night/subjective night.
